# Potential of Adult Endogenous Neural Stem/Progenitor Cells in the Spinal Cord to Contribute to Remyelination in Experimental Autoimmune Encephalomyelitis

**DOI:** 10.3390/cells8091025

**Published:** 2019-09-03

**Authors:** Yuki Maeda, Nami Nakagomi, Akiko Nakano-Doi, Hiroto Ishikawa, Yoshiki Tatsumi, Yoshio Bando, Hiroo Yoshikawa, Tomohiro Matsuyama, Fumi Gomi, Takayuki Nakagomi

**Affiliations:** 1 Department of Ophthalmology, Hyogo College of Medicine, 1-1 Mukogawacho, Nishinomiya, Hyogo 663-8501, Japan (Y.M.) (H.I.) (F.G.); 2 Department of Surgical Pathology, Hyogo College of Medicine, 1-1 Mukogawacho, Nishinomiya, Hyogo 663-8501, Japan; 3 Institute for Advanced Medical Sciences, Hyogo College of Medicine, 1-1 Mukogawacho, Nishinomiya, Hyogo 663-8501, Japan; 4 Department of Therapeutic Progress in Brain Diseases, Hyogo College of Medicine, 1-1 Mukogawacho, Nishinomiya, Hyogo 663-8501, Japan; 5 Division of Neurology, Department of Internal Medicine, Hyogo College of Medicine, 1-1 Mukogawacho, Nishinomiya, Hyogo 663-8501, Japan (Y.T.) (H.Y.); 6 Department of Anatomy, Akita University Graduate School of Medicine, 1-1-1 Hondo, Akita, Akita 010-8543, Japan

**Keywords:** multiple sclerosis, experimental autoimmune encephalomyelitis, neural stem/progenitor cells, Nestin, remyelination, spinal cord

## Abstract

Demyelination and remyelination play pivotal roles in the pathological process of multiple sclerosis (MS) and experimental autoimmune encephalomyelitis (EAE), a well-established animal model of MS. Although increasing evidence shows that various stimuli can promote the activation/induction of endogenous neural stem/progenitor cells (NSPCs) in the central nervous system, the potential contributions of these cells to remyelination following inflammatory injury remain to be fully investigated. In the present study, using an adult mouse model of EAE induced by myelin oligodendrocyte glycoprotein (MOG) peptide, we investigated whether adult NSPCs in the spinal cord can lead to remyelination under inflammatory conditions. Immunohistochemistry showed that cells expressing the NSPC marker Nestin appeared after MOG peptide administration, predominantly at the sites of demyelination where abundant inflammatory cells had accumulated, whereas Nestin^+^ cells were rarely present in the spinal cord of PBS-treated control mice. In vitro, Nestin^+^ NSPCs obtained from EAE mice spinal cords could differentiate into multiple neural lineages, including neurons, astrocytes, and myelin-producing oligodendrocytes. Using the Cre–LoxP system, we established a mouse strain expressing yellow fluorescent protein (YFP) under the control of the *Nestin* promoter and investigated the expression patterns of YFP-expressing cells in the spinal cord after EAE induction. At the chronic phase of the disease, immunohistochemistry showed that YFP^+^ cells in the injured regions expressed markers for various neural lineages, including myelin-forming oligodendrocytes. These results show that adult endogenous NSPCs in the spinal cord can be subject to remyelination under inflammatory conditions, such as after EAE, suggesting that endogenous NSPCs represent a therapeutic target for MS treatment.

## 1. Introduction

Multiple sclerosis (MS), which typically occurs in young adults, is a chronic neuroinflammatory disease of the central nervous system (CNS) [1]. It is pathologically characterized by demyelination, axonal damage, and axonal degeneration due to oligodendrocyte cell lineage damage. Although the precise mechanisms by which MS occurs remain unclear, previous studies have shown that various immune factors, including inflammatory cells (e.g., lymphocytes and macrophages), pro-inflammatory cytokines, and anti-myelin oligodendrocyte glycoprotein (MOG) autoantibodies, are related to the pathogenesis of this disease [1,2,3]. Some patients with MS suffer from various progressive neurological deficits, whereas others occasionally experience improved neurological function during the course of the disease. In experimental autoimmune encephalomyelitis (EAE), both demyelination and remyelination coexist at the same time [4]. Thus, although remyelination is considered responsible for the improvement of clinical symptoms [5,6,7], a detailed mechanism by which remyelination occurs during MS remains unknown.

It is well documented that the CNS, including the brain [8] and the spinal cord [9], harbors multipotent neural stem/progenitor cells (NSPCs) that can produce various types of neural cells, including neurons, astrocytes, and oligodendrocytes. Increasing evidence shows that exogenously transplanted NSPCs can differentiate into myelin-forming oligodendrocytes and improve the functional recovery following CNS injuries [10,11]. Various types of NSPCs (e.g., embryonic stem cells [12], induced pluripotent cells [13], and embryonic CNS cells [14]) have been proposed as a source of cells for treatment. However, cell transplantation using NSPCs has several critical drawbacks, including ethical issues, carcinogenic risk, and complications related to the transplantation procedures. Thus, harnessing the power of endogenous NSPCs seems preferable for the treatment of CNS injuries.

The precise origins and characteristics of NSPCs under pathological conditions still need to be clarified. However, studies have demonstrated that endogenous NSPCs proliferate, migrate, and differentiate after CNS injuries [15,16]. In addition, endogenous NSPCs contribute to neurogenesis in the adult spinal cord in an experimental model of MS [17]. Furthermore, a recent study showed that NSPCs could be isolated from the spinal cord in a mouse model of EAE and that they could differentiate not only into neuronal but oligodendrocyte lineages [18]. These findings suggest that NSPCs induced during EAE have the potential to repair damaged axons through remyelination. However, little is known about the characteristics and fates of endogenous NSPCs in EAE, and it remains unclear as to whether these cells can contribute to remyelination in the context of this disease.

To help clarify these issues, we first examined the spinal cord expression pattern of the NSPC marker Nestin during acute, subacute, and chronic stages of the disease in mice with MOG-induced EAE. In addition, we investigated whether NSPCs isolated from the spinal cord can differentiate into myelin-forming oligodendrocytes. Furthermore, combining genetic lineage labeling of NSPCs against Nestin and the Cre–LoxP system, we examined whether endogenous NSPCs can contribute to myelin regeneration.

## 2. Materials and Methods

### 2.1. Animal Studies

All experimental procedures were approved by the Animal Care Committee of Hyogo College of Medicine (License number: 17-010, 18-026). Adult 6-week-old female C57BL/6 mice (Charles River, Tokyo, Japan) and transgenic mice in the same background strain were used in this study. Nestin-CreERT2 Line4 mice [19] [C57BL/6-Tg(Nes-cre/ERT2)4Imayo mice; RIKEN BioResource Research Center, Ibaragi, Japan] were crossed with yellow fluorescent protein (YFP) reporter mice [B6; 129-Gt(ROSA)26Sortm1(EYFP)/J mice; Jackson Laboratory, Bar Harbor, ME, USA]. The offspring were termed Nestin promoter-driven YFP-expressing (Nestin/YFP) mice. Animals had access to food *ad libitum*, and efforts were made to minimize the number of animals used and their suffering.

### 2.2. Induction and Assessment of EAE

EAE was induced as previously described [20,21], with minor modifications. In brief, C57BL/6 mice or Nestin/YFP mice were immunized subcutaneously with an emulsion containing 200 μg of MOG35-55 (synthesized by Scrum, Tokyo, Japan) and 400 μg of heat-inactivated Mycobacterium tuberculosis (MT) (H37Ra, Difco Laboratories Inc., Detroit, MI, USA) in 100 μL of incomplete Freund’s adjuvant (Sigma-Aldrich, Tokyo, Japan). Zero or 2 days after MOG immunization, mice received 200 ng of pertussis toxin intraperitoneally (Sigma-Aldrich). The development of EAE was assessed daily according to previously defined clinical scores [22,23], with minor modifications: 0, no clinical signs; 1, complete loss of tail tonicity; 2, no hind limb paralysis upon ambulation, but the mouse fails to remain upright when attempted to be rolled by the examiner; 3, paralysis of both hind limbs; 4, paralysis of hind and fore limbs; and 5, death. As soon as the mice reached a clinical score of 4, they were immediately euthanized.

### 2.3. Tamoxifen Treatment

To activate the Cre recombinase, tamoxifen (Toronto Research Chemicals Inc., North York, ON, Canada) was intraperitoneally administrated on 2 consecutive days (3 mg/body/day) in Nestin/YFP mice 4 weeks after MOG immunization. Then, the Nestin^+^ cell fate in EAE mice was evaluated 8 weeks after MOG injection by immunohistochemistry for YFP.

### 2.4. Preparation of Samples from Spinal Cords

C57BL/6 or Nestin/YFP mice were anesthetized with sodium pentobarbital and transcardially perfused with 4% paraformaldehyde (PFA) as described previously [24,25,26]. After fixation in 4% PFA for 24 h, the lumbar cords were carefully removed from the surrounding tissues and processed for paraffin embedding. Samples were then cut into 8 μm sections, followed by hematoxylin and eosin (H&E) staining, luxol fast blue (LFB) staining, and immunohistochemistry. In another experiment, fixed spinal cords were cryoprotected in 30% sucrose, frozen at −80 °C, and cut into 16 μm sections using a cryostat for immunohistochemistry.

### 2.5. Immunohistochemistry

After deparaffinization, sections were subjected to heat treatment by microwave for epitope retrieval in citrate buffer solution (pH 6.0) (Abcam, Cambridge, UK) for 10 min. Then, samples were incubated with primary antibodies against the myelin marker oligodendrocyte-specific protein (OSP) (1:500; rabbit, Abcam), 2′,3′-cyclic-nucleotide 3′-phosphodiesterase (CNPase) (1:1,000; mouse, Abcam), myelin-associated glycoprotein (MAG) (1:2000; mouse, Abcam), CD3 (1:100; rabbit, AnaSpec Inc., San Jose, CA, USA), Nestin (1:100; mouse, Millipore, Temecula, CA, USA), or Ki67 (1:50; mouse, BD Pharmingen, San Diego, CA, USA) at 4 °C for 24 h. Primary antibodies were reacted with secondary antibodies harboring universal immunoperoxidase (N-Histofine Simple Stain Mouse MAX PO, Nichirei Corporation, Tokyo, Japan). Sections were stained by 3,3′-diaminobenzide tetrahydrochloride (DAB, Vector Laboratories Inc., Burlingame, CA, USA), followed by counterstaining with hematoxylin. Images were captured using a microscope (Olympus, Tokyo, Japan).

In separate experiments, spinal cord sections (16 μm thick) were subjected to immunohistochemistry, as previously described [27,28,29,30,31]. In brief, sections were stained using primary antibodies against Sox2 (1:500; rabbit, Millipore), CD31 (1:100; rat, BD Pharmingen), Nestin (1:100; mouse, Millipore), Tuj1 (1:1,000; mouse, Stemcell Technologies, Vancouver, BC, Canada), microtubule-associated protein 2 (MAP2) (1:500; mouse, Sigma-Aldrich), glial fibrillary acidic protein (GFAP) (1:500; mouse, Millipore), Olig2 (1:500; rabbit, Abcam), CNPase (1:200; mouse, Abcam), and MAG (1:200; mouse, Abcam). To detect YFP^+^ cells, an anti-GFP antibody (1:1000; rabbit, Abcam [ab6556]; 1:2000; chicken, Abcam [ab13970]) that recognizes YFP was used. After washing in PBS, sections were stained using Alexa Fluor 488- or 555-conjugated secondary antibodies (1:500; Molecular Probes, Eugene, OR, USA). Cell nuclei were stained with 4′,6-diamidino-2-phenylindole (1:500; DAPI, Kirkegaard & Perry Laboratories, Inc., Gaithersburg, MD, USA). Images were captured using a microscope (Olympus, Tokyo, Japan) and a confocal laser microscope (LSM780; Carl Zeiss AG, Oberkochen, Germany). Primary antibodies were omitted for negative controls, and we confirmed that no staining occurred.

### 2.6. Electron Microscopy (EM)

For EM imaging of lumbar cords, euthanized mice were perfused through the left cardiac ventricle with a fixative containing 1% PFA and 1% glutaraldehyde in phosphate buffer (0.1 M, pH 7.4). EAE or control mice lumbar cords were dissected and fixed by overnight immersion in the same fixative. After washing in PBS, axial thick sections (250 μm) of the lumbar cord tissues cut on a vibratome were fixed in 1% osmium tetroxide for 2 h, rinsed in PBS, dehydrated with a graded ethanol series (70%, 80%, 90%, 95%, and 100%), and embedded in epoxy resin by standard protocol. The resin-embedded tissue was cut into semi-thin (1000 nm) sections, and these were stained with toluidine blue to observe structural details of the lumbar cord axons by light microscopy before ultrathin sectioning. Ultrathin sections (100 nm) were contrasted with uranyl acetate and lead citrate and prepared for electron microscopy imaging to better identify demyelination, using an electron microscope (JEM-1400Plus, JEOL Ltd., Tokyo, Japan).

### 2.7. Cell Cultures

The lumbar cords (L1 and L2 areas) were harvested from EAE mice 4 weeks after MOG peptide administration. As a control, the lumbar cords (L1 and L2 areas) were isolated from control mice 4 weeks after PBS treatment. The tissues were dissected out by passage through 18-, 23-, and 27-gage needles to prepare single-cell suspensions. The resulting cell suspensions were incubated under conditions to promote the proliferation of NSPCs. In brief, they were incubated as adherent cultures using poly-D-lysine-coated six-well plates (Thermo Fisher Scientific, Rochester, NY, USA) in Dulbecco’s Modified Eagle’s Medium containing 20 ng/mL basic fibroblast growth factor (bFGF; PeproTech, Rocky Hill, NJ, USA), 20 ng/mL epidermal growth factor (PeproTech), 1% N_2_ supplement (Invitrogen, Carlsbad, CA, USA), and penicillin (100 U/mL), as previously described [24,26,28,30,31]. On day 18 after incubation, the cells that had adhered were detached with Accutase, and the cells were counted using a hemocytometer. To examine the traits of adherent cells, some were subjected to immunocytochemistry with antibodies against Nestin (1:100; mouse, Millipore) and Sox2 (1:200; rabbit, Millipore). In other experiments, when adherent cells reached confluence, they were detached with Accutase and subsequently subjected to floating culture in a neuron-conditioned medium that promoted the formation of neurosphere-like cell clusters [27,28,29,30]. Then, the cell clusters were subjected to reverse transcription polymerase chain reaction (RT-PCR) (see below) and differentiated on poly-l-lysine-coated (0.05%) glass coverslips for 5 days in neurobasal medium (Invitrogen) containing bFGF, B-27 supplement (Invitrogen), and 2% fetal bovine serum. The differentiated cell clusters were subjected to RT-PCR and immunohistochemistry using antibodies against Tuj1 (1:1000; mouse, Stemcell Technologies), MAP2 (1:1000; rabbit, Millipore), GFAP (1:1000; rabbit, Abcam), proteolipid protein (PLP) (1:500; rabbit, Abcam), myelin basic protein (MBP) (1:500; rabbit, Abcam), and MAG (1:200; mouse, Abcam), followed by Alexa Fluor 488- or 555-conjugated secondary antibodies (1:500; Molecular Probes).

### 2.8. Reverse Transcription Polymerase Chain Reaction

Total RNA was extracted from EAE-induced spinal cord-derived cells using the RNeasy Micro Kit (Qiagen, Hilden, Germany). cDNA was then amplified according to the manufacturer’s protocol [24,32]. The primer sequences used in this study are listed in Table 1.

### 2.9. Statistical Analysis

Data are presented as mean ± standard deviations. Differences were analyzed using Student’s *t*-test. *P* values < 0.05 were considered statistically significant.

## 3. Results

### 3.1. Clinical Deficits in MOG-Induced EAE Mice

**Figure 1:**
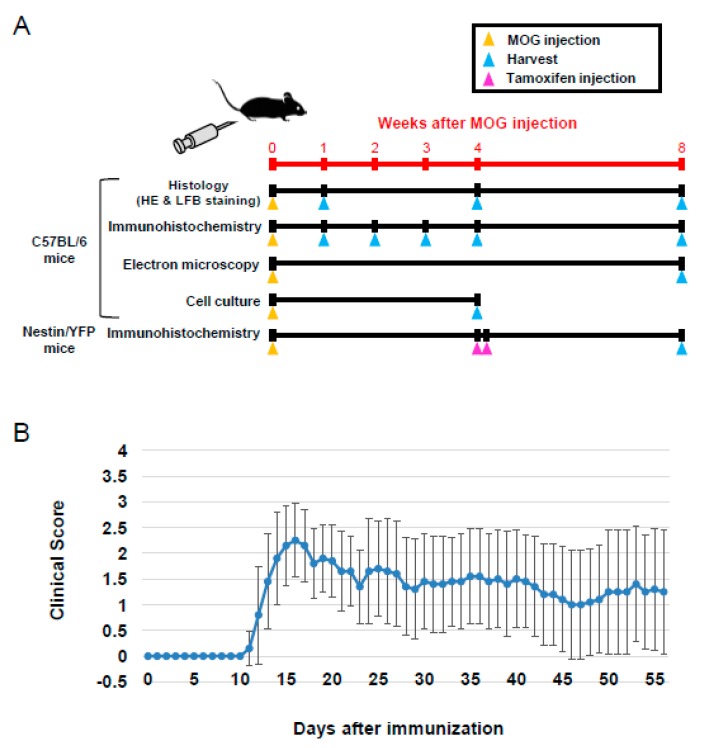
Schematic representation of timing for MOG immunization and tamoxifen injection. Harvested lumbar spinal cords were subjected to histology, immunohistochemistry, EM, and cell culture (**A**). C57BL/6 mice were immunized with MOG, and clinical scores were assessed daily. Results are shown as mean ± SD (*N* = 10) (**B**). Abbreviations: MOG, myelin oligodendrocyte glycoprotein; EM, electron microscopy.

Protocols of this study are summarized in Figure 1A. Clinical scores were assessed in C57BL/6 mice daily for 8 weeks after MOG peptide administration (Figure 1B). The onset of clinical signs appeared 10 days after MOG immunization, and clinical symptoms became more severe approximately 15 days after MOG injection in most of the mice (Figure 1B). Clinical scores of individual mice are shown in Supplemental Table S1. Some mice displayed worsening clinical scores, whereas the scores of others improved (Supplemental Table S1). These data show that the clinical scores of individual mice were variable after the onset of EAE, consistent with the clinical symptoms of MS.

### 3.2. Histopathological Findings in MOG-Induced EAE Mice

**Figure 2:**
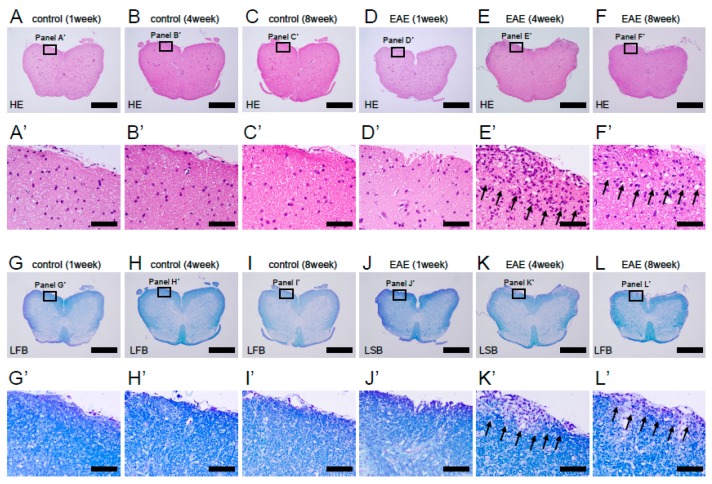
H&E (**A**–**F, A’**–**F’**) and LFB staining (**G–L, G’–L’**) of lumbar spinal cord sections obtained from control (**A**–**C, A’**–**C’, G**–**I,** and **G**’–**I**’) and MOG-immunized mice (**D**–**F**, **D**’–**F**’, **J**–**L**, and **J**’–**L**’) at 1, 4, and 8 weeks after treatment. Infiltration of inflammatory cells and significant demyelination was observed 4 and 8 weeks after treatment in EAE mice, whereas no demyelination was observed at any time points in control mice. Results displayed are representative of three replicates (*N* = 3). Scale bars = 500 µm (**A**–**L**) and 50 µm (**A’**–**L’**). Abbreviations: H&E, hematoxylin and eosin; LFB, luxol fast blue; MOG, myelin oligodendrocyte glycoprotein; EAE, experimental autoimmune encephalomyelitis.

We next investigated histological findings following MOG peptide administration. H&E staining showed that no inflammation was observed at any time point after PBS treatment (1 week after treatment, Figure 2A,A’; 4 weeks after treatment, Figure 2B,B’; and 8 weeks after treatment, Figure 2C,C’). Although inflammatory cells were rarely observed in spinal cords 1 week after MOG peptide administration (Figure 2D,D’), many inflammatory cells, identified morphologically as lymphocytes, were present mainly in the white matter of spinal cords 4 weeks after MOG immunization (Figure 2E,E’). However, such inflammatory responses decreased by 8 weeks after MOG injection (Figure 2F,F’), suggesting that the inflammatory response decreases during the subacute and chronic phases of the disease (i.e., 8 weeks after MOG peptide administration).

Previous studies showed that MOG peptide-induced EAE is characterized by inflammatory changes, but also by spinal cord demyelination. To determine whether our EAE mice experienced demyelination, we performed LFB staining to detect myelin sheath [21,33]. LFB^+^ cells were observed throughout the spinal cord in PBS-treated mice at all time points after treatment (1 week after treatment, Figure 2G,G’; 4 weeks after treatment, Figure 2H,H’; and 8 weeks after treatment, Figure 2I,I’). One week after MOG peptide administration, LFB stain was still present in spinal cords (Figure 2J,J’). However, LFB stain-negative areas were observed in the white matter of spinal cords at 4 (Figure 2K,K’) or 8 weeks after MOG immunization (Figure 2L,L’).

**Figure 3:**
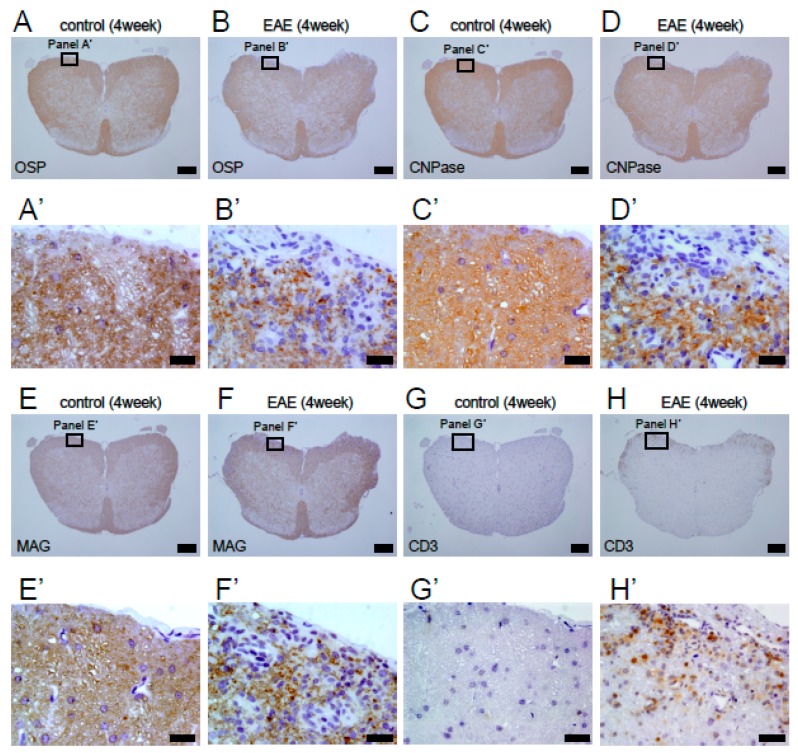
Immunohistochemistry of lumbar spinal cord sections obtained from control (**A**, **A’**, **C**, **C’**, **E**, **E’**, **G**, and **G’**) and MOG-immunized mice (**B**, **B’**, **D**, **D’**, **F**, **F’**, **H**, and **H’**) 4 weeks after treatment. Immunohistochemistry for OSP (**A**, **A’**, **B**, and **B’**), CNPase (**C**, **C’**, **D**, and **D’**), and MAG (**E**, **E’**, **F**, and **F’**) showed that, although control mice widely expressed these oligodendrocyte markers throughout the spinal cord, EAE mice frequency had lost these markers, mainly in the injured white matter. Immunohistochemistry for CD3 showed that, although infiltration of CD3^+^ lymphocytes was rarely observed in control mice (**G**, **G’**), accumulation of many CD3^+^ lymphocytes was observed predominantly in damaged white matter regions in EAE mice (**H**, **H’**). Results displayed are representative of three replicates (*N* = 3). Scale bars = 200 µm (**A**–**H**) and 20 µm (**A’**–**H’**). Abbreviations: MOG, myelin oligodendrocyte glycoprotein; OSP, oligodendrocyte-specific protein; MAG, myelin-associated glycoprotein; EAE, experimental autoimmune encephalomyelitis.

To obtain further evidence of demyelination in EAE mice, spinal cord sections at 4 weeks after MOG injection were subjected to immunohistochemistry with antibodies against oligodendrocyte lineage markers, including OSP, CNPase, and MAG. The results showed that, although OSP^+^ (Figure 3A,A’), CNPase^+^ (Figure 3C,C’), and MAG^+^ cells (Figure 3E,E’) were observed in spinal cords of PBS-treated mice, OSP^+^ (Figure 3B,B’), CNPase^+^ (Figure 3D,D’), and MAG^+^ cells (Figure 3F,F’) were frequently absent in the white matter of EAE mice spinal cords.

Although the precise mechanisms underlying demyelination remain unclear, previous studies have shown that inflammatory cells, such as T lymphocytes, accumulate in the damaged CNS in EAE [7,34] and that T lymphocytes are associated with EAE etiology [35]. Thus, spinal cord sections obtained 4 weeks after MOG immunization were subjected to immunohistochemistry with an antibody against CD3, a pan-T lymphocyte marker. Immunohistochemistry confirmed that, although CD3^+^ lymphocytes were rarely present in PBS-treated mice (Figure 3G,G’), many CD3^+^ lymphocytes were observed in the injured sites of white matter in MOG-injected mice (Figure 3H,H’).

**Figure 4:**
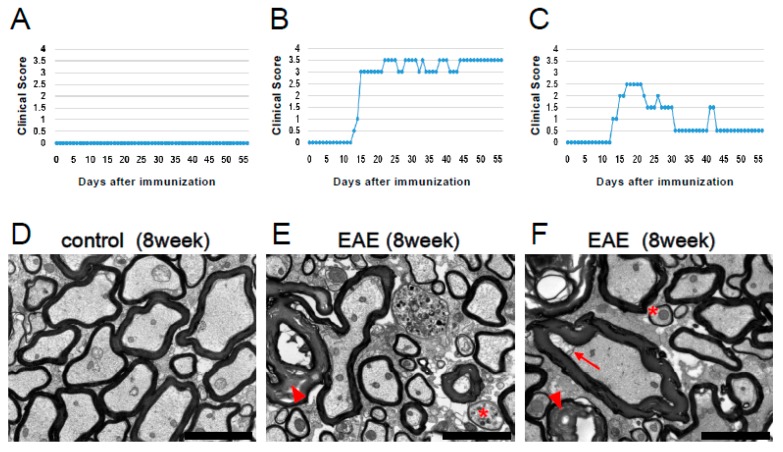
Clinical scores (**A**–**C**) and EM findings (**D**–**F**) in control (**A**, **D**) and MOG-injected EAE mice (**B**, **C**, **E**, and **F**) that either retained high clinical scores (**B**, **E**) or showed an improvement in clinical scores (**C**, **F**). Axons and myelin were intact in the lumbar spinal cord of control mice (**D**). In contrast, both demyelination (excess formation of myelin [arrowheads in **E** and **F**], detachment of myelin from axon [arrow in **F**]) and remyelination (asterisks in **E** and **F**) were observed in lumbar spinal cords after EAE induction. Scale bars = 3 µm (**D**–**F**). Abbreviations: EM, electron microscopy; MOG, myelin oligodendrocyte glycoprotein; EAE, experimental autoimmune encephalomyelitis.

The above data show that inflammatory responses and demyelination occur in MOG-induced EAE mice. However, previous studies have shown that remyelinated nerve fibers, which typically display thin and dense myelin lamellae by EM [4,36], are present in EAE. Thus, we subjected spinal cord samples obtained from MOG-injected mice under EM observation. Control mice displayed no clinical symptoms 8 weeks after PBS injection (Figure 4A), and EM conducted 8 weeks after PBS treatment showed no demyelination or axonal damage (Figure 4D). In contrast, 8 weeks after MOG peptide administration, EAE mice displayed morphological abnormalities of myelin and axon (e.g., excess formation of myelin, detachment of myelin from axon) (Figure 4E,F), which are characteristic traits of demyelination previously described [23,37]. This corresponded with a worsening of clinical scores approximately 12 days after MOG immunization, followed by maintenance of high scores up to 8 weeks (Figure 4B,E), or an improvement of scores (Figure 4C,F). Although more severe damage (e.g., mitochondrial swelling) was frequently observed in EAE mice with consistently elevated clinical scores (Figure 4E), thin-shaped myelin, a reported feature of remyelination [4,36], was present in spinal cords of both groups (Figure 4E,F). These results suggest that mice undergo not only demyelination but also remyelination, during the course of EAE, regardless of whether clinical symptoms improve or not.

### 3.3. Induction of Nestin^+^ NSPCs in MOG-Induced EAE Mice

The aforementioned data suggest that remyelination occurs in MOG-induced EAE mice. The mechanisms responsible for remyelination remain unclear, but increasing evidence shows that NSPCs are induced under various pathological conditions, including EAE [18,20]. Thus, we investigated whether NSPCs, which have the potential to produce cells of various neural lineages, including oligodendrocyte lineages [38,39], are induced in the spinal cord of MOG-injected mice.

**Figure 5:**
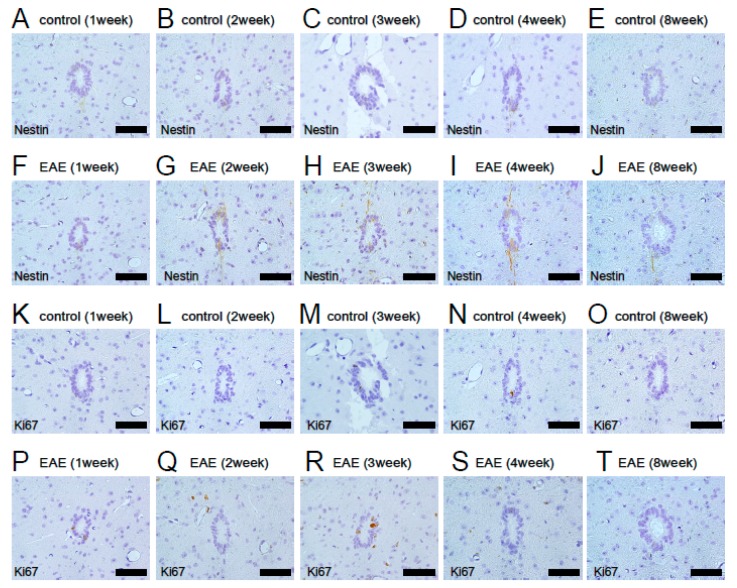
Immunohistochemical analysis for Nestin (**A**–**J**) and Ki67 (**K**–**T**) of central canal ependymal cells at 1 (**A**, **F**, **K**, and **P**), 2 (**B**, **G**, **L**, and **Q**), 3 (**C**, **H**, **M**, and **R**), 4 (**D**, **I**, **N**, and **S**), and 8 weeks (**E**, **J**, **O**, and **T**) after treatment in control (**A**–**E**, **K**–**O**) or EAE mice (**F**–**J**, **P**–**T**). Nestin was rarely expressed in control mice (**A**–**E**) and was slightly increased in EAE mice (**F**–**J**). Ki67^+^ cells were occasionally observed in control (**K**–**O**) and EAE mice (**P**–**T**). However, increased numbers of Ki67^+^ cells were not apparently observed in either group of mice. Results displayed are representative of three replicates (*N* = 3). Scale bars = 50 µm (**A**–**T**). Abbreviations: EAE, experimental autoimmune encephalomyelitis.

Previous studies have shown that ependymal cells around the central canal of the spinal cord display NSPC activities [15,40,41]. We first investigated whether the NSPC marker Nestin is expressed in and around the central canal region. Immunohistochemistry showed that Nestin^+^ cells were rarely observed in central canal ependymal cells in control mice (Figure 5A–E). Although Nestin expression was slightly elevated in EAE mice, it was restricted to central canal regions (Figure 5F–J). In addition, immunohistochemistry for Ki67, a marker of dividing cells, showed that Ki67^+^ proliferative cells were frequently observed in central canal ependymal cells of both control and EAE mice. However, they were not apparently increased in either control (Figure 5K–O) or EAE mice (Figure 5P–T). This suggests that central canal ependymal cells are not a major source of endogenous NSPCs in EAE.

**Figure 6:**
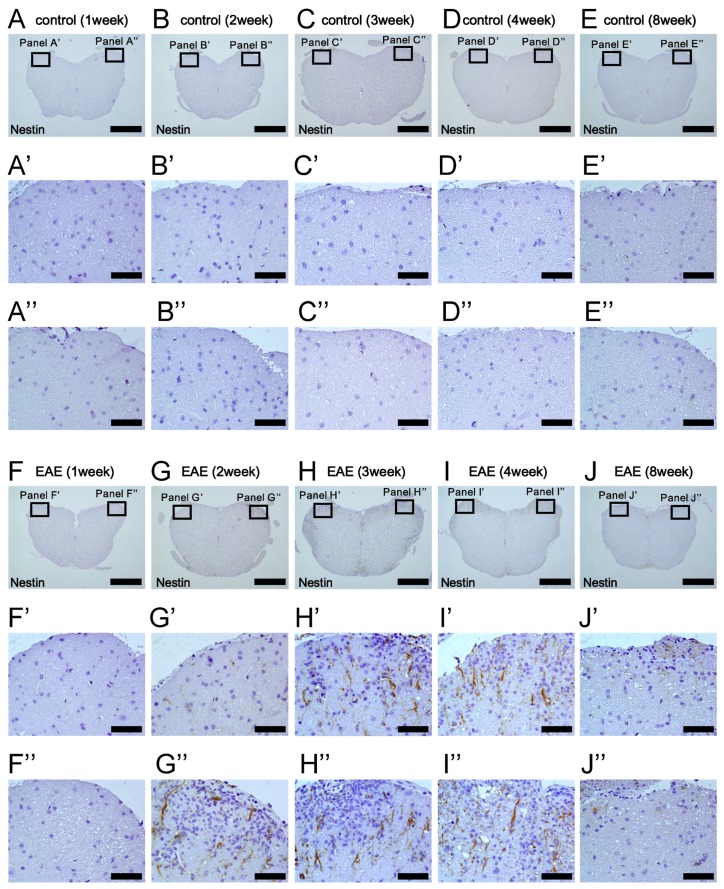
Immunohistochemical analysis for Nestin expression of lumbar spinal cord at 1 (**A**, **A**’, **A’’**, **F**, **F’**, and **F’**’), 2 (**B**, **B’**, **B”**, **G**, **G’**, and **G’’**), 3 (**C**, **C’**, **C’**’, **H**, **H’**, and **H’’**), 4 (**D**, **D’**, **D’’**, **I**, **I’**, and **I’’**), and 8 weeks (**E**, **E’**, **E’’**, **J**, **J’**, and **J’’**) after treatment in control (**A**–**E**, **A’**–**E’**, and **A’’**–**E’’**) and EAE mice (**F**–**J**, **F’**–**J’**, and **F’’**–**J’’**). Although Nestin was rarely expressed in control mice (**A**–**E**, **A’**–**E’**, and **A’**’–**E’’**), many Nestin^+^ cells were observed at 3 (**H**, **H’**, and **H”**) and 4 weeks after MOG injection (**I**, **I’**, and **I”**). Results displayed are representative of three replicates (*N* = 3). Scale bars = 500 µm (**A**–**J**) and 50 µm (**A’**–**J’**, **A”**–**J”**). Abbreviations: EAE, experimental autoimmune encephalomyelitis; MOG, myelin oligodendrocyte glycoprotein.

We next examined the expression pattern of Nestin in white matter areas where demyelination and remyelination were observed following MOG injection. Immunohistochemistry showed that Nestin^+^ cells were not observed in spinal cords after PBS treatment (1 week after treatment, Figure 6A,A’and A’’; 2 weeks after treatment, Figure 6B,B’and B’’; 3 weeks after treatment, Figure 6C,C’ and C’’; 4 weeks after treatment, Figure 6D,D’ and D’’; and 8 weeks after treatment, Figure 6E,E’ and E’’). Nestin^+^ cells were not observed 1 week after MOG injection (Figure 6F,F’ and F’’). However, 2 weeks after MOG immunization, Nestin^+^ cells appeared, predominantly at the injured sites (Figure 6G,G’ and G’’). The numbers of Nestin^+^ cells further increased 3 weeks after MOG injection, and Nestin^+^ cells were widely distributed throughout white matter regions (Figure 6H,H’ and H’’). Although many Nestin^+^ cells were still present 4 weeks after MOG immunization (Figure 6I,I’ and I’’), Nestin expression was decreased 8 weeks after MOG injection (Figure 6J,J’ and J’’). These results show that Nestin expression was induced mainly in the injured areas and peaked 3–4 weeks after MOG peptide administration, indicating that induction of NSPCs occurs during the acute phase of EAE.

**Figure 7:**
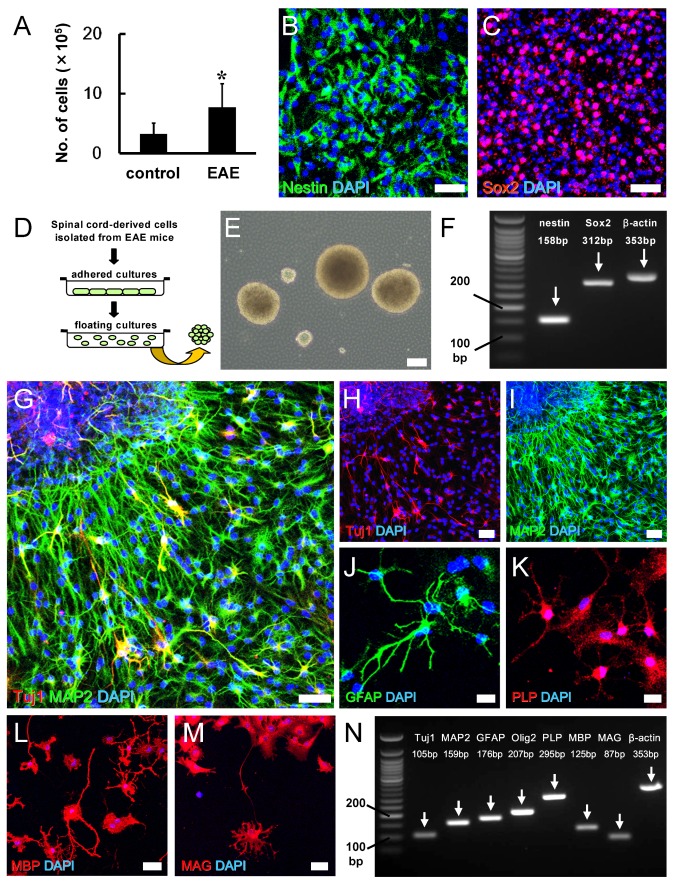
The numbers of adherent cells isolated from spinal cords were significantly higher in EAE mice (*N* = 9) compared with control mice (*N* = 5) (**A**). Immunohistochemistry showed that cells isolated from EAE mice expressed Nestin (Nestin [**B**: green] and DAPI [**B**: blue]) and Sox2 (Sox2 [**C**: red] and DAPI [**C**: blue]). In floating cultures, adherent cells isolated from EAE mice formed neurosphere-like cell clusters (**D**, **E**). PCR analysis also showed that the cell clusters expressed Nestin and Sox2 (**F**). Immunohistochemistry showed that the cell clusters differentiated into Tuj1^+^ (Tuj1 ([**G**, **H**: red] and DAPI [**G**, **H**: blue]) and MAP2^+^ (MAP2 [**G**, **I**: green] and DAPI [**G**, **I**: blue]) neurons, GFAP^+^ astrocytes (GFAP [**J**: green] and DAPI [**J**: blue]), and PLP^+^ (PLP [**K**: red] and DAPI [**K**: blue]), MBP^+^ (MBP [**L**: red] and DAPI [**L**: blue], and MAG^+^ (MAG [**M**: red] and DAPI [**M**: blue] oligodendrocytes. PCR analysis also showed that differentiated cell clusters expressed various neural markers, including for neurons, astrocytes, and oligodendrocytes (**N**). * *P* < 0.05 vs. control. Scale bars = 50 µm (**B**, **C**, **G**, **H**, **I**, **L**, and **M**), 100 µm (**E**), and 20 µm (**J**, **K**). Abbreviations: EAE, experimental autoimmune encephalomyelitis; DAPI, 4′,6-diamidino-2-phenylindole; PCR, polymerase chain reaction; MAP2, microtubule-associated protein 2; GFAP, glial fibrillary acidic protein; PLP, proteolipid protein; MBP, myelin basic protein; MAG, myelin-associated glycoprotein.

We next investigated whether Nestin^+^ cells induced in EAE mice can function as NSPCs that have the potential to differentiate into various neural lineages. Spinal cords were isolated from EAE mice 4 weeks after MOG injection, and resulting cell suspensions were incubated using adherent cultures in medium to promote the proliferation of NSPCs, as described [24,26,28,30,31]. As a control, spinal cords were obtained from PBS-treated mice, and resulting cell suspensions were incubated under the same conditions. Compared with PBS-treated control mice, significantly higher numbers of adherent cells were obtained from cultures derived from MOG-injected EAE mice (Figure 7A). Immunohistochemistry showed that adherent cells largely expressed NSPC markers, including Nestin (Figure 7B) and Sox2 (Figure 7C). Following the incubation in floating cultures (Figure 7D), cells formed neurosphere-like cell clusters (Figure 7E). PCR analysis confirmed that the neurosphere-like cell clusters also expressed Nestin and Sox2 (Figure 7F). After differentiation, they expressed neuronal markers, such as Tuj1 (Figure 7G,H) and MAP2 (Figure 7G,I), the astrocyte marker GFAP (Figure 7J), and oligodendrocyte markers, such as PLP (Figure 7K), MBP (Figure 7L), and MAG (Figure 7M). Similarly, PCR analysis showed that differentiated cells expressed markers for neurons (Tuj1, MAP2), astrocytes (GFAP), and oligodendrocyte lineages (Olig2, PLP, MBP, and MAG) (Figure 7N).

### 3.4. Potential for Remyelination Induced by NSPCs in EAE

The above data indicate that Nestin^+^ NSPCs were induced in EAE mice and that they could differentiate into various neural lineages, including oligodendrocyte lineages, *in vitro*. Although the numbers of adherent cells, which likely included NSPC cells, were higher in EAE mice compared with control mice, they were also found in control mice. Thus, it was unclear whether Nestin^+^ NSPCs induced at the sites of damage contribute to remyelination following EAE *in vivo*.

**Figure 8:**
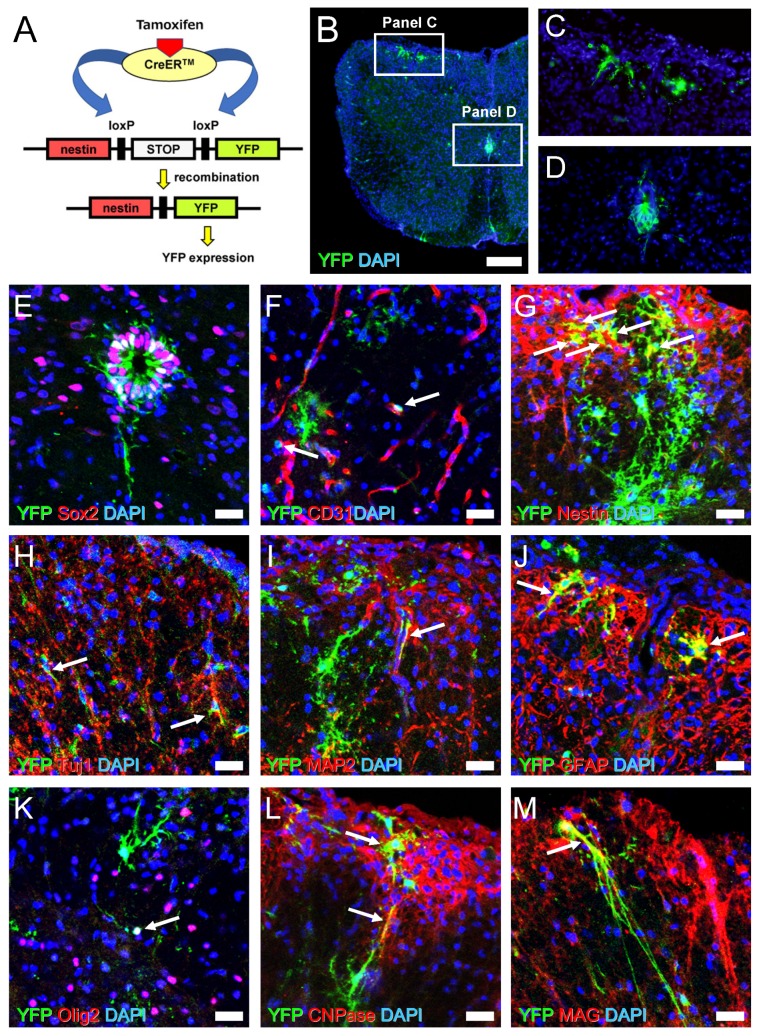
Schematic representation for recombination by tamoxifen injection in Nestin/YFP mice (**A**). Immunohistochemistry showed that YFP^+^ cells are observed in white matter (**B**, **C**) and central canal ependymal cells (**B**, **D**) (YFP [**B**–**D**: green] and DAPI [**B**–**D**: blue]). Immunohistochemistry of central canal areas showed that some ependymal cells expressed both YFP and Sox2 (YFP [**E**: green], Sox2 [**E**: red], and DAPI [**E**: blue]). Immunohistochemistry of white matter showed YFP^+^ cells localized near CD31^+^ blood vessels (YFP [**F**: green], CD31 [**F**: red], and DAPI [**F**: blue]) (arrows). Immunohistochemistry of white matter showed some YFP^+^ cells co-expressed Nestin (YFP [**G**: green], Nestin [**G**: red], and DAPI [**G**: blue]) (arrows), Tuj1 (YFP [**H**: green], Tuj1 [**H**: red], and DAPI [**H**: blue]) (arrows), MAP2 (YFP [**I**: green], MAP2 [**I**: red], and DAPI [**I**: blue]) (arrows), GFAP (YFP [**J**: green], GFAP [**J**: red], and DAPI [**J**: blue]) (arrows), Olig2 (YFP [**K**: green], Olig2 [**K**: red], and DAPI [**K**: blue]) (arrows), CNPase (YFP [**L**: green], CNPase [**L**: red], and DAPI [**L**: blue]) (arrows), and MAG (YFP [**M**: green], MAG [**M**: red], and DAPI [**M**: blue]) (arrows). Results displayed are representative of three replicates (*N* = 3). Scale bars = 200 µm (**B**) and 25 µm (**E**–**M**). Abbreviations: YFP, yellow fluorescent protein; DAPI, 4′,6-diamidino-2-phenylindole; MAP2, microtubule-associated protein 2; GFAP, glial fibrillary acidic protein; CNPase, 2′,3′-cyclic-nucleotide 3′-phosphodiesterase; MAG, myelin-associated glycoprotein.

To clarify this, we established a strain of mice expressing YFP under the control of the *Nestin* promoter (Figure 8A). Tamoxifen was administrated in Nestin/YFP mice 4 weeks after MOG immunization because Nestin is highly expressed at that time (Figure 6I,I’ and I’’). Four weeks after tamoxifen induction (8 weeks after MOG treatment), spinal cord-derived sections were stained with an anti-GFP antibody that can also detect YFP [25].

Immunohistochemistry showed that YFP^+^ cells were observed mainly in white matter areas of EAE mice spinal cords (Figure 8B,C). YFP^+^ cells were also detectable in ependymal cells around the central canal (Figure 8B,D). Although some YFP^+^ ependymal cells showed expression of the NSPC marker Sox2 (Figure 8E), YFP^+^ ependymal cells were restricted to the central canal even 8 weeks after MOG immunization (Figure 8B,D,E). These findings indicate that YFP^+^ cells in white matter regions are likely derived from regionally induced NSPCs rather than ependymal cells from the central canal.

Although the precise origin of locally induced Nestin^+^ NSPCs in EAE remains unclear, previous studies showed that various types of NSPCs reside near blood vessels [31,42,43]. Our immunohistochemical analyses showed that some YFP^+^ cells, particularly with immature round shapes, localized near CD31^+^ endothelial cells in white matter regions (Figure 8F). Although some YFP^+^ cells still expressed Nestin, differentiated YFP^+^ cells largely did not express Nestin (Figure 8G). This suggests that most YFP^+^ cells have already lost the characteristic traits of NSPCs and differentiated into mature neural cells.

To confirm this hypothesis, we investigated whether YFP^+^ cells express mature neural cell markers. We found that some YFP^+^ cells observed in white matter regions differentiated into neurons (Tuj1^+^ [Figure 8H] and MAP2^+^ cells [Figure 8I]) and GFAP^+^ astrocytes (Figure 8J). We also investigated whether YFP^+^ cells could differentiate into oligodendrocyte lineage cells. We found that some YFP^+^ cells with immature round forms expressed Olig2, an oligodendrocyte precursor cell (OPC) marker (Figure 8K), suggesting that NSPCs differentiated into OPCs. In addition, some differentiated YFP^+^ cells expressed mature oligodendrocyte markers, including CNPase (Figure 8L) and MAG (Figure 8M). These results show that Nestin^+^ NSPCs induced in EAE mice can differentiate into mature oligodendrocytes in vivo, presumably in part through OPCs, indicating that they could contribute to remyelination during the course of MS.

## 4. Discussion

This study demonstrates for the first time that NSPCs are induced mainly at injured areas of the spinal cord in a mouse model of EAE and that these NSPCs have the potential to differentiate into myelin-producing oligodendrocytes *in vitro* and *in vivo*. These results suggest that remyelination is induced in the damaged spinal cord during the course of MS.

A previous study showed that the number of Nestin^+^ NSPCs was the smallest in the lumbar segment, compared with the cervical and thoracic segments, in spinal cords of adult mice [44]. In this study, however, we found that many Nestin^+^ NSPCs emerged at the injured regions of the lumbar spinal cord, in concurrence with severe inflammatory changes following MOG immunization. These results strongly suggest that NSPCs are induced in response to spinal cord injuries. In support of this hypothesis, using a mouse model of cerebral infarction, we have previously demonstrated that Nestin^+^ NSPCs, which had the potential to generate neurons, astrocytes, and oligodendrocytes, were specifically induced within post-stroke areas, whereas these cells were not induced within contralateral non-ischemic areas [27,30]. In addition, we previously showed that oxygen-glucose deprivation, which mimics hypoxia/ischemia *in vivo*, could promote the induction of stem cell populations [24,45]. However, severe hypoxia/ischemia does not likely occur during the course of MS. Alternatively, previous studies showed that inflammatory cytokines (e.g., TNF alpha, interleukin 1 beta) exert positive effects on NSPCs (e.g., cell proliferation) [46,47]. Thus, inflammation mediators (e.g., inflammatory cytokines) may be major activators of NSPCs under pathological conditions of MS. However, the precise mechanisms by which NSPCs are induced in EAE should be addressed by further studies.

The brain comprises many types of stem cells, including NSPCs in the subventricular zone (SVZ) [42,43], reactive astrocytes [48,49], ependymal cells [50], radial glia-like cells [51], OPCs [52,53], NG^+^ glia [54,55], and ischemia/injury-induced stem cells presumably derived from brain pericytes following ischemia [26,32,56,57]. Although the myelin reparative potentials of these cells have not been fully investigated, previous studies using the Cre–LoxP system demonstrated that SVZ-derived NSPCs and OPCs could contribute to myelin regeneration in mouse brains following demyelination [58,59].

In the spinal cord, several types of stem cells, including OPCs [60], radial glia [61], and ependymal cells [15,40,41,62], have been proposed. Among them, it is reported that only ependymal cells are multipotent stem cells that can generate all types of neural cells, including neurons, astrocytes, and oligodendrocytes [15,40,41,62]. A previous study showed that ependymal NSPCs migrate toward inflammatory areas and differentiate into neuronal cells [17]. However, a recent study clearly showed that ependymal NSPCs fail to migrate toward damaged regions and remain near the central canal after spinal cord injury [63]. In addition, a separate study showed that, although central canal ependymal cells proliferate in response to spinal cord injuries, they do not proliferate following EAE [64]. Similar to these studies, we could not obtain evidence that central canal ependymal cells were significantly proliferative in response to demyelination following MOG injection. These findings strongly indicate that ependymal cells are not a major source of endogenous NSPCs in EAE. To confirm this finding, it is essential to determine if cells isolated from EAE model spinal cords, excluding the central canal regions, have traits of NSPCs. Unfortunately, because of the methodological difficulties of selectively removing tissues around the central canal, we could not perform that experiment in this study. However, consistent with a previous report [63], our current study also showed that, although YFP^+^ cells were observed in ependymal cells around the central canal after EAE, they were restricted within these areas, even at chronic phases. In contrast, many YFP^+^ cells emerged at the demyelinated injured areas. These results strongly indicate that locally induced NSPCs, rather than ependymal cells around the central canal, likely function as stem cells and contribute to regenerate the injured regions during EAE.

In this study, we found that some YFP^+^ cells with immature round shapes were present near endothelial cells in white matter regions, consistent with previous reports showing that several types of stem cell populations, such as NSPCs in the SVZ [42,43], radial glia-like cells [65], OPCs [66,67], and reactive pericytes [24,30,31], reside near blood vessels. Taken together, these results suggest that NSPCs may originate, at least in part, from cells localized in perivascular regions (e.g., pericytes) of injured white matter, as we proposed previously [24,30,31]. However, to confirm this, fate mapping studies using lineage-specific marker for pericytes (e.g., PDGFRβ, NG2, and αSMA) are required.

Although the precise origin of NSPCs during EAE remains unclear, previous reports showed that OPCs also contribute to myelin regeneration in mouse spinal cords after demyelination [60]. Thus, some myelin regeneration might be attributable to OPCs. It has been reported that Nestin is expressed in early stages of oligodendrocyte lineages [68] and that OPCs are developmentally derived from NSPCs [69]. Consistent with these reports, we found that some YFP^+^ NSPCs, which originally stained positive for Nestin, expressed the OPC marker Olig2. This suggests that NSPCs contribute to myelin regeneration, at least in part, through OPCs. However, the precise relationship between NSPCs and OPCs remains unclear, and whether OPCs directly contribute to remyelination during EAE should be determined by lineage-specific fate mapping for OPCs (e.g., Olig2, NG2, and PDGFRα).

During the clinical course of EAE, we found that the neurological deficits of some mice improved. Although the underlying mechanisms remain unclear, EM showed that thin-shaped myelin in the spinal cord of EAE mice. In addition, fate mapping using Nestin/YFP mice showed that YFP^+^ cells gave rise to cells expressing the myelin protein. These results suggest that functional improvement following EAE can in part be attributed to the reparative processes of remyelination. In support of this view, previous studies demonstrated that transplantation of NSPCs into injured spinal cords improved neurological deficits through their differentiation into myelin-producing oligodendrocytes [10,11,13]. Furthermore, previous studies showed that NSPCs transplanted into injured spinal cords differentiated into neurons and exhibited functional recovery [12,14]. Consistent with these studies, we found that YFP^+^ cells at the white matter near the anterior horn gave rise to neuronal cells, as well as myelin-forming oligodendrocytes. As the anterior horn is the place where upper motor neurons and lower motor neurons are connected through synapses, neurogenesis around the area might also be associated with functional improvement.

In the present study, the clinical scores of some mice improved following the acute phase of EAE, whereas clinical scores of others remained poor. To understand whether remyelination indeed contributes to functional recovery, it would be necessary to investigate whether the numbers of YFP^+^ cells that co-express myelin proteins (e.g., MBP, MAG) are higher in the EAE mice that displayed improved clinical scores versus the EAE mice whose scores remained the same or worsened.

Currently, we have not investigated whether NSPCs induced in EAE mice predominantly differentiate into specific lineages (e.g., neuronal, astrocytic, or oligodendrocyte lineages) *in vivo* and *in vitro*. However, previous studies showed that several factors, such as extracellular matrix, Wnt/beta-catenin signaling, and microRNAs could regulate the fate of NSPCs [62,70,71]. Thus, in terms of promoting remyelination, it is essential to identify the factors that control the fate of NSPCs and/or promote the differentiation toward specific lineages (e.g., oligodendrocyte lineages).

In conclusion, we show here that NSPCs are regionally induced in the spinal cord in response to injuries associated with a mouse model of EAE. As these cells have the capacity to regenerate myelin, developing factors that regulate the cell survival and proliferation of NSPCs and/or promote their differentiation toward oligodendrocyte lineages could be useful for accelerating myelin regeneration in patients with MS.

## Figures and Tables

**Table 1:** List and sequence of mouse primers used for RT-PCR analysis.

Primers	Sequence (5′→3′) (F: Forward; R: Reverse)	Size
β-actin	F: GCTCGTCGTCGACAAGGGCTC; R: CAAACATGATCTGGGTCATCTTCTC	353bp
GFAP	F: TCGGCCAGTTACCAGGAGG; R: ATGGTGATGCGGTTTTCTTCG	176bp
MAG	F: CAAGTCCCGCACACAAGTG; R: AGCAGGGTACAGTTTCGTAGG	87bp
MAP2	F: CTCATTCGCTGAGCCTTTAGAC; R: ACTGGAGGCAACTTTTCTCCT	159bp
MBP	F: TCACAGCGATCCAAGTACCTG; R: CCCCTGTCACCGCTAAAGAA	125bp
Nestin	F: CGCTGGAACAGAGATTGGAAG; R: CATCTTGAGGTGTGCCAGTT	158bp
Olig2	F: TGGAGAGATGCGTTCGTTCC; R: GTGCTCTGCGTCTCGTCTAA	207bp
PLP	F: TGAGCGCAACGGTAACAGG; R: GGGAGAACACCATACATTCTGG	295bp
Sox2	F: TTGGGAGGGGTGCAAAAAGA; R: CCTGCGAAGCGCCTAACGTA	312bp
Tuj1	F: TGAGGCCTCCTCTCACAAGT; R: GGCCTGAATAGGTGTCCAAA	105bp

## Supplementary Materials

The following are available online at https://www.mdpi.com/2073-4409/8/9/1025/s1. Table S1: Clinical scores of individual mice after MOG immunization.
