# Supplementary material for: Potential of Adult Endogenous Neural Stem/Progenitor Cells in the Spinal Cord to Contribute to Remyelination in Experimental Autoimmune Encephalomyelitis
Source: Cells. 2019 Sep 3;8(9):1025. doi: 10.3390/cells8091025 (PMC6769975; doi:10.3390/cells8091025)
Supplement: Supplementary file 1 [file cells-08-01025-s001.pdf]

Supplementary Table 1.
Clinical scores of individual mice after MOG immunization (N = 10)

	No.1	No.2	No.3	No.4	No.5	No.6	No.7	No.8	No.9	No.10
**Day0**	0	0	0	0	0	0	0	0	0	0
**Day1**	0	0	0	0	0	0	0	0	0	0
**Day2**	0	0	0	0	0	0	0	0	0	0
**Day3**	0	0	0	0	0	0	0	0	0	0
**Day4**	0	0	0	0	0	0	0	0	0	0
**Day5**	0	0	0	0	0	0	0	0	0	0
**Day6**	0	0	0	0	0	0	0	0	0	0
**Day7**	0	0	0	0	0	0	0	0	0	0
**Day8**	0	0	0	0	0	0	0	0	0	0
**Day9**	0	0	0	0	0	0	0	0	0	0
**Day10**	0	0	0	0	0	0	0	0	0	0
**Day11**	1	0	0	0	0.5	0	0	0	0	0
**Day12**	3	1	0	0	1	0.5	0	0	1.5	1
**Day13**	3	2	0	1.5	1	1.5	1.5	0	2	2
**Day14**	3	2	0	2	2	2	1.5	1	3	2.5
**Day15**	3	2	0.5	1.5	2.5	3	2	2	3	2
**Day16**	3	2	1	1.5	2	3	2	3	3	2
**Day17**	3	1.5	1	1.5	2	3	2	2.5	3	2
**Day18**	3	1.5	1	1	1.5	2.5	2	1.5	2.5	1.5
**Day19**	2.5	1	1	1.5	1.5	2	2.5	2	3	2
**Day20**	2.5	1	1	1	2	2	2.5	1.5	3	2
**Day21**	2	1.5	0.5	0.5	2	2	2	1	3	2
**Day22**	2	1	0.5	1.5	2	1.5	1.5	1.5	3	2
**Day23**	2	1	0.5	1	1	1	1.5	1	3	1.5
**Day24**	3	0.5	0.5	3	1	1	2	1	3	1.5
**Day25**	3	1	1	3	1	1	1.5	1	3	1.5
**Day26**	3	0.5	1	3	1	1	2	0.5	3	1.5
**Day27**	3	0.5	1	3	1	1	1.5	0.5	3	1.5
**Day28**	3	0.5	1	1	1.5	0.5	1.5	1	3	0.5
**Day29**	3	0.5	0.5	1	1.5	0.5	1.5	1	3	0.5
**Day30**	3	0.5	0.5	1	1.5	1	2	1	3	1

**Day31**	3	0.5	0.5	1.5	1.5	1	1.5	1	3	0.5
**Day32**	3	0.5	0.5	1	1.5	1	1.5	1.5	3	0.5
**Day33**	3	1	0.5	1.5	1.5	1	1	1	3	1
**Day34**	3	0.5	0.5	1.5	2	1	1	1	3	1
**Day35**	3	0.5	0.5	2	2	1	1.5	1	3	1
**Day36**	3	0.5	0.5	2	2	1	1.5	1	3	1
**Day37**	3	0.5	0.5	2	1.5	1	1	1	3	1
**Day38**	3	0.5	1	2.5	1	1	1	1	3	1
**Day39**	3	0.5	1	2.5	1	0.5	1	1	3	0.5
**Day40**	3	0.5	1	2.5	1	1	1	1	3	1
**Day41**	3	0.5	1	2	1	1	1	1	3	1
**Day42**	3	0.5	1	2	0.5	1	1	1	3	0.5
**Day43**	3	0.5	1	1	1	0.5	0.5	1	3	0.5
**Day44**	3	0.5	0.5	1	1	0.5	1	1	3	0.5
**Day45**	3	0.5	1	1	0.5	0.5	0.5	0.5	3	0.5
**Day46**	3	0.5	0.5	0.5	0.5	0.5	0.5	0.5	3	0.5
**Day47**	3	0.5	0.5	0.5	0.5	0.5	0.5	0.5	3	0.5
**Day48**	3	0.5	1	0.5	0.5	0.5	0.5	0.5	3	0.5
**Day49**	3	0.5	1.5	0.5	0.5	0.5	0.5	0.5	3	0.5
**Day50**	3	0.5	3	0.5	0.5	0.5	0.5	0.5	3	0.5
**Day51**	3	0.5	3	0.5	0.5	0.5	0.5	0.5	3	0.5
**Day52**	3	0.5	3	0.5	0.5	0.5	0.5	0.5	3	0.5
**Day53**	3	0.5	3	1	0.5	0.5	1	1	3	0.5
**Day54**	3	0.5	2.5	0.5	0.5	0.5	0.5	1	3	0.5
**Day55**	3	0.5	3	0.5	0.5	0.5	0.5	1	3	0.5
**Day56**	3	0.5	3	0.5	0.5	0.5	0.5	0.5	3	0.5
